# Complete Genome Sequence of the Fruiting Myxobacterium *Melittangium boletus* DSM 14713

**DOI:** 10.1128/genomeA.01262-17

**Published:** 2017-11-09

**Authors:** Anke Treuner-Lange, Marc Bruckskotten, Oliver Rupp, Alexander Goesmann, Lotte Søgaard-Andersen

**Affiliations:** a Department of Ecophysiology, Max Planck Institute for Terrestrial Microbiology, Marburg, Germany; b Bioinformatics and Systems Biology, Justus-Liebig University Giessen, Giessen, Germany

## Abstract

The formation of spore-filled fruiting bodies in response to starvation represents a hallmark of many members of the order *Myxococcales*. Here, we present the complete 9.9-Mb genome of the fruiting type strain *Melittangium boletus* DSM 14713, the first member of this genus to have its genome sequenced.

## GENOME ANNOUNCEMENT

In response to starvation, most members of the order *Myxococcales* initiate a developmental program that culminates in the formation of multicellular spore-filled fruiting bodies (1, 2). Interestingly, comparative genome investigations using genomes from eight different genera of the *Myxococcales* have indicated that the developmental program that ultimately results in fruiting body formation may not be highly conserved (3–5). Currently, the order *Myxococcales* consists of 3 suborders, with 55 species from 28 genera (6). So far, 21 complete and 36 draft *Myxococcales* genome sequences are available (3, 7–32), representing 18 different genera. Of the 10 genera without any genome data available, 3 genera (*Pyxidicoccus*, *Aggregicoccus*, and *Melittangium*) belong to the suborder *Cystobacterineae*.

The model organism *Myxococcus xanthus*, a member of the suborder *Cystobacterineae*, has been extensively studied to investigate the genetic basis underlying fruiting body formation (33, 34). To generate additional resources for accurate genome comparisons as well as to understand the evolution of the genetic program for fruiting body formation, we sequenced and annotated the complete genome of *Melittangium boletus* DSM 14713, which was obtained from the Deutsche Sammlung von Mikroorganismen und Zellkulturen GmbH.

After confirming fruiting body formation with the formation of sporangioles on slime stalks by *M. boletus* DSM 14713, we collected genomic DNA (35) and sequenced it using PacBio single-molecule real-time (SMRT) sequencing (36) on the PacBio RSII platform at the Max Planck-Genome-Centre, Cologne, Germany. Three SMRT cells were used. Additionally, 16,186,722 100-bp paired-end Illumina reads were obtained using the HiSeq 2000 platform. After quality evaluation and filtering of 184,213 subreads, the assembly process using the hierarchical genome-assembly process (HGAP) assembly pipeline (37) resulted in one contig with an 83-fold coverage. This contig was inspected by YASS (Yet Another Similarity Searcher) (38) and manually closed using the Gepard dotplot generator (39), together with the Illumina reads and the Pilon tool (40), and finally oriented to DnaA as the first locus tag. The genome annotation was prepared using Prokka (41). BLASTP searches against the RefSeq database were used to assign functional annotation and identify possible frameshifts in genes. The corresponding genes were removed from the annotation.

The complete genome sequence of *M. boletus* DSM 14713 contains 9,910,441 bp, with a GC content of 68.4%. A total of 8,018 protein-coding sequences (CDSs) were identified, together with 69 tRNA genes and 12 rRNA operons. The size of the *M. boletus* genome is similar to those of other genomes of fruiting myxobacteria, which range in size from 9.0 Mb to 16.0 Mb. Aligning the *M. boletus* genome with other completely sequenced *Myxococcales* genomes using NUCmer (42) revealed that the genomes of *Archangium gephyra* DSM 2261 and *Stigmatella aurantiaca* DW4/3-1 most closely matched, with 39.8% and 16.8% of the sequences aligning, respectively.

The *M. boletus* genome sequence is the first sequence of this species and genus and will provide an important resource to delineate the genetic determinants involved in fruiting body formation and shared by members of the *Cystobacterineae* and possibly also by other *Myxococcales* outside this suborder.

### Accession number(s).

The genome sequence was deposited in GenBank under accession number CP022163.
